# Supplementary material for: Task-related, intrinsic oscillatory and aperiodic neural activity predict performance in naturalistic team-based training scenarios
Source: Sci Rep. 2022 Sep 28;12:16172. doi: 10.1038/s41598-022-20704-8 (PMC9519541; doi:10.1038/s41598-022-20704-8)
Supplement: Supplementary file 1 — Supplementary Information. [file 41598_2022_20704_MOESM1_ESM.pdf]

Table S1: Summary of model produced by the call `lmer(formula = power ~ role * session * study + Months_Current + (1 | ch_name) + (1 | dyad/subj), data = tf_power_theta, REML = TRUE, control = lmerControl(optimizer = "bobyqa", calc.derivs = TRUE))`
Linear mixed model fit by REML. t-tests use Satterthwaite's method

REML criterion at convergence: 502

Scaled residuals:

Min	1Q	Median	3Q	Max
-4.01	-0.63	-0.02	0.57	4.16

Random effects:

Groups	Term	Std.Dev.
subj:dyad	(Intercept)	0.42042
ch_name	(Intercept)	0.10895
dyad	(Intercept)	0.20491
Residual		0.24842

Number of obs: 2658, groups: subj:dyad, 39; ch$_{n}ame, 25; dyad, 20$.

Fixed effects:

	Estimate	Std. Error	df	t value	Pr(>|t|)	
(Intercept)	−7.7	0.13	34	−58	1.7e−35	***
role[Commander]	0.27	0.08	21	3.4	0.0029	**
session[Low]	−0.0073	0.0071	2.6e+03	−1	0.31	
session[Med]	−0.047	0.0071	2.6e+03	−6.7	2.4e−11	***
study[Armoured]	−0.21	0.083	17	−2.6	0.019	*
Months$_{C}urrent$	−0.0011	0.0025	31	−0.44	0.66	
role[Commander]:session[Low]	−0.12	0.0071	2.6e+03	−17	3.1e−59	***
role[Commander]:session[Med]	0.05	0.0071	2.6e+03	7.1	1.5e−12	***
role[Commander]:study[Armoured]	0.011	0.068	16	0.16	0.88	
session[Low]:study[Armoured]	0.035	0.0071	2.6e+03	4.9	9.9e−07	***
session[Med]:study[Armoured]	0.061	0.0071	2.6e+03	8.6	1.4e−17	***
role[Commander]:session[Low]:study[Armoured]	−0.062	0.0071	2.6e+03	−8.6	1e−17	***
role[Commander]:session[Med]:study[Armoured]	0.053	0.0071	2.6e+03	7.5	7.1e−14	***

Table S2: Summary of model produced by the call `lmer(formula = power ~ role * session * study + Months_Current + (1 | ch_name) + (1 | dyad/subj), data = tf_power_alpha, REML = TRUE, control = lmerControl(optimizer = "bobyqa", calc.derivs = TRUE))`
Linear mixed model fit by REML. t-tests use Satterthwaite's method

REML criterion at convergence: -1325

Scaled residuals:

Min	1Q	Median	3Q	Max
-3.24	-0.62	-0.06	0.59	3.68

Random effects:

Groups	Term	Std.Dev.
subj:dyad	(Intercept)	0.267492
ch_name	(Intercept)	0.086437
dyad	(Intercept)	0.179752
Residual		0.174597

Number of obs: 2595, groups: subj:dyad, 38; ch$_{n}ame, 25; dyad, 20$.

Fixed effects:

	Estimate	Std. Error	df	t value	Pr(>|t|)	
(Intercept)	−8.8	0.094	32	−93	1.7e−40	***
role[Commander]	0.19	0.052	20	3.6	0.0019	**
session[Low]	0.031	0.0051	2.5e+03	6.2	6e−10	***
session[Med]	−0.034	0.005	2.5e+03	−6.7	3e−11	***
study[Armoured]	−0.046	0.06	18	−0.76	0.46	
Months$_{C}urrent$	−0.0019	0.0017	31	−1.2	0.26	
role[Commander]:session[Low]	−0.068	0.0051	2.5e+03	−13	6.3e−40	***
role[Commander]:session[Med]	0.034	0.005	2.5e+03	6.7	2.9e−11	***
role[Commander]:study[Armoured]	−0.074	0.044	17	−1.7	0.11	
session[Low]:study[Armoured]	0.021	0.0051	2.5e+03	4.2	2.9e−05	***
session[Med]:study[Armoured]	0.044	0.005	2.5e+03	8.8	2.2e−18	***
role[Commander]:session[Low]:study[Armoured]	−0.02	0.0051	2.5e+03	−4	6.6e−05	***
role[Commander]:session[Med]:study[Armoured]	0.031	0.005	2.5e+03	6.1	1.3e−09	***

Table S3: Summary of model produced by the call `lmer(formula = individual_rating ~ power * role * session + Months_Current + (1 | dyad/subj), data = dome_theta, REML = TRUE, control = lmerControl(optimizer = "bobyqa", calc.derivs = TRUE))`
Linear mixed model fit by REML. t-tests use Satterthwaite's method

REML criterion at convergence: 2629

Scaled residuals:

Min	1Q	Median	3Q	Max
-2.75	-0.7	-0.02	0.77	3.09

Random effects:

Groups	Term	Std.Dev.
subj:dyad	(Intercept)	0.34027
dyad	(Intercept)	1.03068
Residual		0.55880

Number of obs: 1486, groups: subj:dyad, 20; dyad, 10.

Fixed effects:

	Estimate	Std. Error	df	t value	Pr(>|t|)	
(Intercept)	9.9	0.58	66	17	6.3e−26	***
power	0.33	0.057	1.3e+03	5.8	9.1e−09	***
role[Commander]	−0.53	0.44	4.1e+02	−1.2	0.23	
session.L	−1.7	0.38	1.5e+03	−4.4	1.3e−05	***
session.Q	0.23	0.47	1.5e+03	0.48	0.63	
$\text{Months}_{Current}$	0.0015	0.0051	8.7	0.29	0.78	
power:role[Commander]	−0.068	0.057	9.7e+02	−1.2	0.23	
power:session.L	−0.19	0.051	1.5e+03	−3.7	0.00021	***
power:session.Q	0.018	0.061	1.5e+03	0.3	0.77	
role[Commander]:session.L	3.1	0.38	1.5e+03	8	2.6e−15	***
role[Commander]:session.Q	−0.14	0.47	1.5e+03	−0.3	0.76	
power:role[Commander]:session.L	0.42	0.051	1.5e+03	8.2	5.3e−16	***
power:role[Commander]:session.Q	0.007	0.061	1.5e+03	0.11	0.91	

Table S4: Summary of model produced by the call `lmer(formula = individual_rating ~ power * role * session + Months_Current + (1 | dyad/subj), data = dome_alpha, REML = TRUE, control = lmerControl(optimizer = "bobyqa", calc.derivs = TRUE))`
Linear mixed model fit by REML. t-tests use Satterthwaite's method

REML criterion at convergence: 2694

Scaled residuals:

Min	1Q	Median	3Q	Max
-2.87	-0.72	0	0.6	2.85

Random effects:

Groups	Term	Std.Dev.
subj:dyad	(Intercept)	0.37189
dyad	(Intercept)	1.02906
Residual		0.57578

Number of obs: 1474, groups: subj:dyad, 20; dyad, 10.

Fixed effects:

	Estimate	Std. Error	df	t value	Pr(>|t|)	
(Intercept)	9.1	0.78	1.8e+02	12	9.5e−24	***
power	0.2	0.078	1.2e+03	2.6	0.01	*
role[Commander]	−0.21	0.69	6.7e+02	−0.3	0.76	
session.L	−1.4	0.66	1.5e+03	−2.2	0.028	*
session.Q	−1.2	0.71	1.5e+03	−1.6	0.1	
$\text{Months}_{Current}$	0.0027	0.0056	8.9	0.48	0.64	
power:role[Commander]	−0.024	0.077	1e+03	−0.31	0.76	
power:session.L	−0.14	0.074	1.5e+03	−1.9	0.059	.
power:session.Q	−0.13	0.079	1.5e+03	−1.7	0.092	.
role[Commander]:session.L	3	0.66	1.5e+03	4.5	6.8e−06	***
role[Commander]:session.Q	3.1	0.71	1.5e+03	4.3	1.6e−05	***
power:role[Commander]:session.L	0.35	0.074	1.5e+03	4.7	2.7e−06	***
power:role[Commander]:session.Q	0.37	0.079	1.5e+03	4.6	4.3e−06	***

Table S5: Summary of model produced by the call `lmer(formula = Score ~ power * role * session + Months_Current + (1 | dyad/subj), data = Tank_Final_Alpha, REML = TRUE, control = lmerControl(optimizer = "bobyqa", calc.derivs = TRUE))`
Linear mixed model fit by REML. t-tests use Satterthwaite's method

REML criterion at convergence: 3007

Scaled residuals:

Min	1Q	Median	3Q	Max
-2.87	-0.67	-0.09	0.74	2.7

Random effects:

Groups	Term	Std.Dev.
subj:dyad	(Intercept)	6.6975
dyad	(Intercept)	28.1459
Residual		17.8546

Number of obs: 351, groups: subj:dyad, 16; dyad, 9.

Fixed effects:

	Estimate	Std. Error	df	t value	Pr(>|t|)	
(Intercept)	5.2e+02	49	2.8e+02	11	1.9e−22	***
power	8.3	5.4	3.1e+02	1.5	0.12	
role[Commander]	2e+02	43	59	4.6	2.1e−05	***
session.L	80	59	3.3e+02	1.4	0.17	
session.Q	86	56	3.3e+02	1.5	0.13	
Months$_{Current}$	0.022	0.094	6	0.24	0.82	
power:role[Commander]	22	4.8	59	4.7	1.5e−05	***
power:session.L	10	6.6	3.3e+02	1.6	0.11	
power:session.Q	8.4	6.3	3.3e+02	1.3	0.18	
role[Commander]:session.L	−3.7e+02	58	3.2e+02	−6.4	4.9e−10	***
role[Commander]:session.Q	1.5e+02	56	3.3e+02	2.7	0.0071	**
power:role[Commander]:session.L	−41	6.5	3.2e+02	−6.4	5.7e−10	***
power:role[Commander]:session.Q	17	6.3	3.3e+02	2.7	0.007	**

Table S6: Summary of model produced by the call `lmer(formula = Score ~ power * role * session + Months_Current + (1 | dyad/subj), data = Tank_Final_Theta, REML = TRUE, control = lmerControl(optimizer = "bobyqa", calc.derivs = TRUE))`
Linear mixed model fit by REML. t-tests use Satterthwaite's method

REML criterion at convergence: 3069

Scaled residuals:

Min	1Q	Median	3Q	Max
-2.93	-0.63	-0.05	0.61	2.99

Random effects:

Groups	Term	Std.Dev.
subj:dyad	(Intercept)	3.1451
dyad	(Intercept)	35.0842
Residual		18.7539

Number of obs: 354, groups: subj:dyad, 17; dyad, 9.

Fixed effects:

	Estimate	Std. Error	df	t value	Pr(>|t|)	
(Intercept)	4.7e+02	31	84	15	1.4e−25	***
power	4.6	3.7	1.1e+02	1.2	0.21	
role[Commander]	27	25	20	1.1	0.28	
session.L	1.9e+02	39	2.9e+02	4.9	1.4e−06	***
session.Q	0.55	37	3.2e+02	0.015	0.99	
$\text{Months}_{Current}$	0.13	0.06	5.6	2.1	0.082	.
power:role[Commander]	3.9	3.1	20	1.3	0.22	
power:session.L	25	4.9	3e+02	5.2	3.5e−07	***
power:session.Q	−1.2	4.6	3.2e+02	−0.27	0.79	
role[Commander]:session.L	−1.4e+02	38	2.2e+02	−3.6	0.00033	***
role[Commander]:session.Q	−48	37	3.3e+02	−1.3	0.19	
power:role[Commander]:session.L	−17	4.7	2.2e+02	−3.5	0.00053	***
power:role[Commander]:session.Q	−6	4.6	3.3e+02	−1.3	0.19	

Table S7: Summary of model produced by the call `lmer(formula = power ~ role * performer * study * session + Months_Current + (1 | ch_name) + (1 | dyad/subj), data = composite_theta, REML = TRUE, control = lmerControl(optimizer = "bobyqa", calc.derivs = TRUE))`
Linear mixed model fit by REML. t-tests use Satterthwaite's method

REML criterion at convergence: 216

Scaled residuals:

Min	1Q	Median	3Q	Max
-4.21	-0.61	0.02	0.61	4.28

Random effects:

Groups	Term	Std.Dev.
subj:dyad	(Intercept)	0.49267
ch_name	(Intercept)	0.11432
dyad	(Intercept)	0.14800
Residual		0.23089

Number of obs: 2483, groups: subj:dyad, 37; $\text{ch}_{n}ame, 25; dyad, 19$.

Fixed effects:

	Estimate	Std. Error	df	t value	Pr(>|t|)	
(Intercept)	−7.8	0.15	30	−52	5.6e−31	***
role[Commander]	0.18	0.099	21	1.9	0.078	.
performer[High]	−0.00096	0.008	2.4e+03	−0.12	0.9	
study[Armoured]	−0.27	0.089	15	−3	0.0091	**
session[Low]	−0.049	0.0088	2.4e+03	−5.5	4.6e−08	***
session[Med]	−0.054	0.0081	2.4e+03	−6.6	4e−11	***
$\text{Months}_{C}urrent$	0.00045	0.0029	32	0.15	0.88	
role[Commander]:performer[High]	0.014	0.008	2.4e+03	1.8	0.072	.
role[Commander]:study[Armoured]	−0.056	0.082	15	−0.68	0.51	
performer[High]:study[Armoured]	−0.016	0.008	2.4e+03	−2	0.042	*
role[Commander]:session[Low]	−0.17	0.0088	2.4e+03	−19	3.4e−77	***
role[Commander]:session[Med]	0.072	0.0081	2.4e+03	8.9	1.1e−18	***
performer[High]:session[Low]	0.087	0.0096	2.4e+03	9.1	2.8e−19	***
performer[High]:session[Med]	−0.043	0.0085	2.4e+03	−5	5.3e−07	***
study[Armoured]:session[Low]	−0.0005	0.0088	2.4e+03	−0.057	0.95	
study[Armoured]:session[Med]	0.051	0.0081	2.4e+03	6.3	2.8e−10	***
role[Commander]:performer[High]:study[Armoured]	0.0091	0.008	2.4e+03	1.1	0.26	
role[Commander]:performer[High]:session[Low]	0.13	0.0096	2.4e+03	13	6.5e−39	***
role[Commander]:performer[High]:session[Med]	−0.00087	0.0085	2.4e+03	−0.1	0.92	
role[Commander]:study[Armoured]:session[Low]	−0.11	0.0088	2.4e+03	−12	6.9e−33	***
role[Commander]:study[Armoured]:session[Med]	0.069	0.0081	2.4e+03	8.5	2.3e−17	***
performer[High]:study[Armoured]:session[Low]	0.075	0.0096	2.4e+03	7.8	6.4e−15	***
performer[High]:study[Armoured]:session[Med]	−0.047	0.0085	2.4e+03	−5.5	3.9e−08	***
role[Commander]:performer[High]:study[Armoured]:session[Low]	0.13	0.0096	2.4e+03	14	2e−42	***
role[Commander]:performer[High]:study[Armoured]:session[Med]	−0.056	0.0085	2.4e+03	−6.5	8.3e−11	***

Table S8: Summary of model produced by the call `lmer(formula = power ~ role * performer * study * session + Months_Current + (1 | ch_name) + (1 | dyad/subj), data = composite_alpha, REML = TRUE, control = lmerControl(optimizer = "bobyqa", calc.derivs = TRUE))`
Linear mixed model fit by REML. t-tests use Satterthwaite's method

REML criterion at convergence: -1442

Scaled residuals:

Min	1Q	Median	3Q	Max
-3.18	-0.59	-0.03	0.59	3.92

Random effects:

Groups	Term	Std.Dev.
subj:dyad	(Intercept)	0.270293
ch_name	(Intercept)	0.087913
dyad	(Intercept)	0.190125
Residual		0.163479

Number of obs: 2420, groups: subj:dyad, 36; ch$_{n}ame, 25; dyad, 19$.

Fixed effects:

	Estimate	Std. Error	df	t value	Pr(>|t|)	
(Intercept)	−8.9	0.1	29	−88	3.5e−37	***
role[Commander]	0.1	0.057	19	1.8	0.09	.
performer[High]	−0.0011	0.0057	2.4e+03	−0.19	0.85	
study[Armoured]	−0.062	0.064	17	−0.97	0.35	
session[Low]	0.0073	0.0063	2.4e+03	1.1	0.25	
session[Med]	−0.04	0.0058	2.3e+03	−6.9	5.5e−12	***
Months$_{C}urrent$	−0.00017	0.0018	30	−0.093	0.93	
role[Commander]:performer[High]	0.035	0.0057	2.4e+03	6.1	9.4e−10	***
role[Commander]:study[Armoured]	−0.13	0.046	16	−2.8	0.012	*
performer[High]:study[Armoured]	−0.005	0.0057	2.4e+03	−0.89	0.37	
role[Commander]:session[Low]	−0.11	0.0063	2.4e+03	−18	3e−67	***
role[Commander]:session[Med]	0.058	0.0058	2.3e+03	10	1.9e−23	***
performer[High]:session[Low]	0.029	0.0069	2.4e+03	4.2	2.4e−05	***
performer[High]:session[Med]	−0.031	0.0061	2.3e+03	−5.1	4.4e−07	***
study[Armoured]:session[Low]	−0.0048	0.0063	2.3e+03	−0.76	0.45	
study[Armoured]:session[Med]	0.037	0.0058	2.3e+03	6.3	2.7e−10	***
role[Commander]:performer[High]:study[Armoured]	0.028	0.0057	2.4e+03	4.9	1.2e−06	***
role[Commander]:performer[High]:session[Low]	0.076	0.0069	2.4e+03	11	1.8e−27	***
role[Commander]:performer[High]:session[Med]	−0.011	0.0061	2.3e+03	−1.8	0.078	.
role[Commander]:study[Armoured]:session[Low]	−0.068	0.0063	2.3e+03	−11	2.4e−26	***
role[Commander]:study[Armoured]:session[Med]	0.051	0.0058	2.3e+03	8.8	3e−18	***
performer[High]:study[Armoured]:session[Low]	0.038	0.0069	2.4e+03	5.6	3.1e−08	***
performer[High]:study[Armoured]:session[Med]	−0.029	0.0061	2.3e+03	−4.7	2.9e−06	***
role[Commander]:performer[High]:study[Armoured]:session[Low]	0.11	0.0069	2.4e+03	16	2.2e−53	***
role[Commander]:performer[High]:study[Armoured]:session[Med]	−0.039	0.0061	2.3e+03	−6.4	2.1e−10	***

Table S9: Summary of effects of model examining theta across role, session and study

	Chisq	Df	Pr(>Chisq)
role	10.8641542	1	0.0009804
session	117.2068625	2	0.0000000
study	6.4677887	1	0.0109847
Months_Current	0.1915799	1	0.6616049
role:session	237.6790886	2	0.0000000
role:study	0.0141432	1	0.9053347
session:study	191.4389080	2	0.0000000
role:session:study	88.7225461	2	0.0000000

Table S10: Summary of effects of model examining alpha across role, session and study

	Chisq	Df	Pr(>Chisq)
role	12.9050171	1	0.0003277
session	93.7484328	2	0.0000000
study	0.5474413	1	0.4593651
Months_Current	1.3230171	1	0.2500513
role:session	165.2905681	2	0.0000000
role:study	2.8583450	1	0.0909010
session:study	172.6115801	2	0.0000000
role:session:study	38.4953238	2	0.0000000

Table S11: Summary of effects of theta model on behaviour at GBAD

	Chisq	Df	Pr(>Chisq)
power	29.8304947	1	0.0000000
role	0.0017186	1	0.9669327
session	46.4531177	2	0.0000000
Months_Current	0.0851113	1	0.7704867
power:role	0.2825403	1	0.5950410
power:session	34.3994751	2	0.0000000
role:session	44.3711766	2	0.0000000
power:role:session	68.1091969	2	0.0000000

Table S12: Summary of effects of alpha model on behaviour at GBAD

	Chisq	Df	Pr(>Chisq)
power	8.0346420	1	0.0045891
role	0.0251982	1	0.8738741
session	39.7234743	2	0.0000000
Months_Current	0.2314303	1	0.6304653
power:role	0.8235653	1	0.3641398
power:session	1.1520049	2	0.5621411
role:session	56.9775739	2	0.0000000
power:role:session	39.3305173	2	0.0000000

Table S13: Summary of effects of alpha model on behaviour at Armoured

	Chisq	Df	Pr(>Chisq)
power	1.2749098	1	0.2588476
role	0.6653663	1	0.4146718
session	91.1723292	2	0.0000000
Months_Current	0.0576444	1	0.8102585
power:role	28.4933789	1	0.0000001
power:session	18.8508083	2	0.0000806
role:session	1.0706166	2	0.5854887
power:role:session	44.7294316	2	0.0000000

Table S14: Summary of effects of theta model on behaviour at Armoured

	Chisq	Df	Pr(>Chisq)
power	3.234076	1	0.0721210
role	3.545752	1	0.0596981
session	66.453656	2	0.0000000
Months_Current	4.468449	1	0.0345263
power:role	2.499945	1	0.1138502
power:session	41.185424	2	0.0000000
role:session	7.223879	2	0.0269994
power:role:session	14.594387	2	0.0006774

Table S15: Summary of GAMM for alpha power predicting behaviour at GBAD

Predictors	Estimate	S.E.	t	p
Intercept	7.38	0.11	65.00	<.001
Role[Commander]	-0.33	0.11	-2.92	.004
Smoothers	e.d.f.	d.f.	F	p
ti(power)	9.429	4	4.12	<.001
ti(power):RoleCommander	3.542	4	11.05	<.001
ti(power):RoleGunner	5.459	4	0.00	.45
s(channel)	5.100	7	0.00	.99
Adjusted R^2	.28			
Observations	160			

Note. For GAMMS, predictors and smoothers are treatment coded, with commanders set as the reference level for Role. e.d.f. = estimated degrees of freedom (associated p values are only approximate); ti() = tensor product interaction term; s() = by-channel factor smooth.

Table S16: Summary of GAMM for theta power predicting behaviour at GBAD

Predictors		Estimate	S.E.	t	p
Intercept		7.29	0.13	54.22	<.001
Role[Commander]		-0.12	0.13	-0.93	.35
Smoothers		e.d.f.	d.f.	F	p
ti(power)		1.231	4	0.00	.02
ti(power):RoleCommander		2.702	4	4.71	<.001
ti(power):RoleGunner		1.647	4	1.38	.03
s(channel)		6.434	5	0.00	.99
Adjusted R^2	.17				
Observations	120				

Table S17: Summary of GAMM for aperiodic slope predicting behaviour at GBAD

Predictors		Estimate	S.E.	t	p
Intercept		7.46	0.10	68.99	<.001
Role[Commander]		-0.09	0.10	-0.83	.40
Smoothers		e.d.f.	d.f.	F	p
ti(slope)		7.558	4	0.00	.06
ti(slope):RoleCommander		1.583	4	0.87	.10
ti(slope):RoleGunner		2.216	4	6.12	<.001
s(channel)		6.427	7	0.00	.99
Adjusted R^2	.15				
Observations	160				

Table S18: Summary of GAMM for aperiodic intercept predicting behaviour at GBAD

Predictors		Estimate	S.E.	t	p
Intercept		7.38	0.10	68.64	<.001
Role[Commander]		-0.09	0.10	-0.09	.92
Smoothers		e.d.f.	d.f.	F	p
ti(intercept)		9.324	4	3.44	<.001
ti(intercept):RoleCommander		2.033	4	4.28	<.001
ti(intercept):RoleGunner		1.504	4	0.87	.08
s(channel)		5.760	7	0.00	.99
Adjusted R^2	.17				
Observations	160				

Table S19: Summary of general linear model for behaviour predicting behaviour at GBAD

Predictor	b	b 95% CI [LL, UL]	sr^2	sr^2 95% CI [LL, UL]	Fit
(Intercept)	1.11	[-4.06, 6.28]			
individual_rating_Low	0.83*	[0.16, 1.50]	.29	[-.04, .63]	
role[Commander]	-2.46	[-7.63, 2.71]	.04	[-.11, .19]	
individual_rating_Low:role[Commander]	0.31	[-0.37, 0.98]	.04	[-.10, .18]	
					R^2 = .31

Note. For general linear models, a significant b-weight indicates the semi-partial correlation is also significant. b represents unstandardized regression weights. sr^2 represents the semi-partial correlation squared. LL and UL indicate the lower and upper limits of a confidence interval, respectively. * indicates $p < .05$. ** indicates $p < .01$.

Table S20: Summary of general linear model for IAF predicting behaviour at GBAD

Predictor	b	b 95% CI [LL, UL]	sr^2	sr^2 95% CI [LL, UL]	Fit
(Intercept)	17.68*	[4.13, 31.23]			
cog	-1.02	[-2.38, 0.34]	.14	[-.14, .42]	
role[Commander]	-4.92	[-18.47,8.63]	.03	[-.11, .18]	
cog:role[Commander]	0.47	[-0.89, 1.83]	.03	[-.11, .17]	
					R^2 = .17

Table S21: Summary of GAMM for alpha power predicting behaviour at Armoured

Predictors	Estimate	S.E.	t	p
Intercept	441.25	2.65	165.98	<.001
Role[Commander]	-6.55	2.66	-2.46	.01
Smoothers	e.d.f.	d.f.	F	p
ti(power)	1.493	4	2.38	.002
ti(power):RoleCommander	2.463	4	4.22	<.001
ti(power):RoleGunner	5.710	4	0.00	.10
s(channel)	2.707	7	0.00	.99
Adjusted R^2	.26			
Observations	80			

Table S22: Summary of GAMM for theta power predicting behaviour at Armoured

Predictors		Estimate	S.E.	t	p
Intercept		441.51	2.84	155.51	<.001
Role[Commander]		-7.09	2.87	-2.46	.01
Smoothers		e.d.f.	d.f.	F	p
ti(power)		8.598	4	1.53	.006
ti(power):RoleCommander		2.517	4	5.61	<.001
ti(power):RoleGunner		8.686	4	0.57	.09
s(channel)		5.255	5	0.00	.84
Adjusted R^2	.42				
Observations	60				

Table S23: Summary of GAMM for aperiodic intercept predicting behaviour at Armoured

Predictors		Estimate	S.E.	t	p
Intercept		442.40	2.36	187.01	<.001
Role[Commander]		-6.73	2.37	-2.83	.005
Smoothers		e.d.f.	d.f.	F	p
ti(intercept)		9.386	4	3.81	.006
ti(intercept):RoleCommander		6.944	4	0.32	.15
ti(intercept):RoleGunner		5.582	4	0.00	.87
s(channel)		2.862	7	0.00	.91
Adjusted R^2	.24				
Observations	96				

Table S24: Summary of GAMM for aperiodic slope predicting behaviour at Armoured

Predictors		Estimate	S.E.	t	p
Intercept		442.25	2.05	215.38	<.001
Role[Commander]		-7.65	2.15	-3.54	<.001
Smoothers		e.d.f.	d.f.	F	p
ti(slope)		2.983	4	15.61	.001
ti(slope):RoleCommander		3.437	4	0.00	.66
ti(slope):RoleGunner		8.256	4	0.00	.38
s(channel)		1.356	7	0.00	.62
Adjusted R^2	.42				
Observations	96				

Table S25: Summary of general linear model for behaviour predicting behaviour at Armoured

Predictor	b	b 95% CI [LL, UL]	sr^2	sr^2 95% CI [LL, UL]	Fit
(Intercept)	412.26**	[232.58, 591.93]			
Score_1	0.07	[-0.33, 0.47]	.01	[-.09, .11]	
role[Commander]	-4.30	[-183.97, 175.37]	.00	[-.01, .01]	
Score_1:role[Commander]	0.02	[-0.38, 0.41]	.00	[-.02, .02]	
					R^2 = .01

Table S26: Summary of general linear model for IAF predicting behaviour at Armoured

Predictor	b	b 95% CI [LL, UL]	sr^2	sr^2 95% CI [LL, UL]	Fit
(Intercept)	1057.63**	[672.82, 1442.44]			
cog	-61.89**	[-101.01, -22.76]	.44	[.08, .79]	
role[Commander]	234.63	[-150.18, 619.44]	.07	[-.10, .23]	
cog:role[Commander]	-24.22	[-63.35, 14.91]	.07	[-.10, .24]	
					R^2 = .55*

Table S27. Summary of the mixed-effects beta regression model.

Family: beta (logit)

Formula: variance ~ study * role * session + (1 | dyad/subj)

AIC	BIC	logLik	deviance	df.resid
-416.5	-376.3	223.3	-446.5	93

Random effects:

Conditional model

Groups	Name	Variance	Std.Dev.
subj:dyad	(Intercept)	0.007	0.08
dyad	(Intercept)	0.0003	0.02

Number of obs: 108, groups: subj:dyad, 39; dyad, 20

Dispersion parameter for beta family (): 247

Conditional model:

	Estimate	Std.Error	z value	p
Intercept	-1.15	0.02	-55.69	<.001
study[Armoured]	0.02	0.02	0.94	0.35
role[Commander]	-0.01	0.02	-0.81	0.41
session[Low]	0.03	0.02	1.89	0.06
session[Med]	0.02	0.02	1.25	0.21
study[Armoured]:role[Commander]	0.01	0.02	0.81	0.41
study[Armoured]:session[Low]	-0.006	0.02	-0.31	0.75
study[Armoured]:session[Med]	-0.005	0.02	-0.27	0.78
role[Commander]:session[Low]	0.02	0.02	0.88	0.38
role[Commander]:session[Med]	0.003	0.02	0.17	0.86
study[Armoured]:role[Commander]:session[Low]	-0.01	0.02	-0.67	0.50
study[Armoured]:role[Commander]:session[Med]	-0.002	0.02	-0.11	0.91

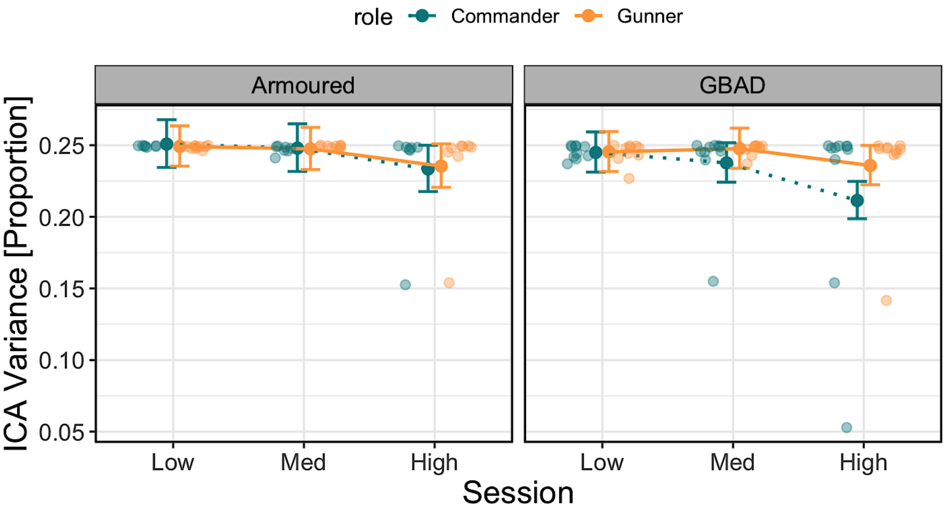

Figure S1. Modelled effects of the proportion of variance explained by EOG components from the independent component analysis. (A) Variance explained (y-axis; higher values indicate more proportion of variance explained) across scenario one (Low), two (Med) and three (High; x-axis) between Armoured (left) and GBAD (right). Estimates from commanders are represented by the dashed blue line, while estimates from gunners are represented by the solid orange line. Error bars represent the 83% confidence interval. Individual dots represent raw single-subject data points.
